# Trait-Specific Responses of Wild Bee Communities to Landscape Composition, Configuration and Local Factors

**DOI:** 10.1371/journal.pone.0104439

**Published:** 2014-08-19

**Authors:** Sebastian Hopfenmüller, Ingolf Steffan-Dewenter, Andrea Holzschuh

**Affiliations:** Department of Animal Ecology and Tropical Biology, Biocenter, University of Würzburg, Würzburg, Germany; Institute of Botany, Czech Academy of Sciences, Czech Republic

## Abstract

Land-use intensification and loss of semi-natural habitats have induced a severe decline of bee diversity in agricultural landscapes. Semi-natural habitats like calcareous grasslands are among the most important bee habitats in central Europe, but they are threatened by decreasing habitat area and quality, and by homogenization of the surrounding landscape affecting both landscape composition and configuration. In this study we tested the importance of habitat area, quality and connectivity as well as landscape composition and configuration on wild bees in calcareous grasslands. We made detailed trait-specific analyses as bees with different traits might differ in their response to the tested factors. Species richness and abundance of wild bees were surveyed on 23 calcareous grassland patches in Southern Germany with independent gradients in local and landscape factors. Total wild bee richness was positively affected by complex landscape configuration, large habitat area and high habitat quality (i.e. steep slopes). Cuckoo bee richness was positively affected by complex landscape configuration and large habitat area whereas habitat specialists were only affected by the local factors habitat area and habitat quality. Small social generalists were positively influenced by habitat area whereas large social generalists (bumblebees) were positively affected by landscape composition (high percentage of semi-natural habitats). Our results emphasize a strong dependence of habitat specialists on local habitat characteristics, whereas cuckoo bees and bumblebees are more likely affected by the surrounding landscape. We conclude that a combination of large high-quality patches and heterogeneous landscapes maintains high bee species richness and communities with diverse trait composition. Such diverse communities might stabilize pollination services provided to crops and wild plants on local and landscape scales.

## Introduction

Global food security and stable ecosystem services like pollination are major challenges that the fast growing human population has to deal with in the next decades [1], [2]. Agricultural landscapes where remaining natural and semi-natural habitats are often highly fragmented and degraded [3] suffer from loss of pollinators and increasing effects of global change pressures [4], [5]. Therefore the conservation of pollinating insects should be a major issue for providing pollination services to agricultural and natural ecosystems [6], [7]. Wild bees are one of the most important pollinator groups [8] and their diversity can influence pollination services [9], [10]. In agroecosystems themselves pollinators can be negatively influenced by isolation from semi-natural habitats [11], [12]. Still there is little knowledge how wild bee diversity can be enhanced in semi-natural habitats to provide a high and stable spillover of wild bees to agroecosystems and secure pollination of insect-pollinated plants. Land-use change is considered the major driver of global biodiversity change [13], and therefore understanding patterns and driving factors of wild bee diversity in agricultural landscapes is an essential precondition for maintaining stable ecosystems and crop pollination worldwide.

One of the most species rich but highly fragmented habitats in central Europe are calcareous grasslands, that are in severe decline since the middle of the 19^th^ century [3]. This is due to the decrease of historical land-use such as shepherding, as well as forestation and fertilization. Through the severe loss of these habitats many of the remaining fragments are strongly isolated and many species specialized on these habitats are threatened [14]. This shows that the identification of factors that influence species diversity, especially of habitat specialists on these habitat patches is important for conservation and restoration. The relative importance of different factors influencing species richness and population viability, like local factors (e.g. habitat area and quality) and landscape factors (eg. landscape composition, landscape configuration and habitat connectivity) are still controversially discussed or unclear [15]–[18]. This might be because different factors could affect different life-history traits of bees and therefore trait-specific analysis are helpful to disentangle the importance of these factors. The different definition and use of traits has led to some confusion, especially in plant ecology [19]. Therefore new approaches have been developed and the use of trait based measures like community weighted mean and functional diversity have been proposed [20]. Nevertheless, in studies dealing with wild bee diversity the use of life-history traits usually based on categories is a widely used approach (e.g. [12], [21], [22]). Community shifts can be identified by using community weighted mean [23] but this might overlook species groups that share different traits and are smaller in numbers (e.g. small social bees [24]). As bee diversity effects on pollination can be driven by functional complementarity [10], knowledge about trait-specific performance of bees are also needed to preserve pollination services that should consequently only sufficiently provided by a combination of bee-traits.

Despite the often shown positive species-area relationship [25], [26] and the widespread notion that size matters, several studies showed that habitat quality can be even more important for insect populations than habitat area [27]–[29]. In wild bees habitat quality includes both quality of food (pollen and nectar diversity) and of nesting resources (mainly sun exposed soil in central Europe), whereby quality of nesting resources has rarely been tested (but see [30]). Habitat area and quality thus seem to be important factors for insect diversity but it is still unclear how important the heterogeneity of the surrounding landscape is [18]. This question should be addressed by separating the effects of landscape composition and configuration that are both expected to influence species diversity in habitat patches [17]. Landscape composition - particularly the percentage of semi-natural habitats in the landscape - has been shown to affect insect diversity [31], [32]. In contrast, effects of landscape configuration - for example edge or patch density - that are independent of effects of landscape composition have rarely been tested [17] because these are often confounding factors (landscapes with high amount of semi-natural habitat are often also highly structured, but see [33], [34]). Landscape configuration can be measured in different ways and the most simple is patch density, i.e. the number of patches in the landscape [34]. The re-allocation of agricultural land causing lower patch densities is still a threat to edge habitats that provide resources and can promote dispersal [35], [36]. A recent meta-analysis found only weak effects of landscape configuration on wild bee diversity in agroecosystems [12] but most of the studies included in this analysis were not explicitly designed to focus on landscape configuration. There are still no studies focusing on the effects of landscape configuration in semi-natural habitats, but as linear landscape structures and edge habitats are important foraging and nesting resources for wild pollinators [36], there is a strong need to understand how important the landscape configuration is for pollinator communities.

While landscape configuration describes the arrangement of patches in the landscape independently of habitat type, habitat connectivity describes the areas and arrangement of habitat patches (e.g. of calcareous grasslands) in the landscape around a focal habitat patch. High connectivity of calcareous grasslands has been shown to positively affect butterflies and plants ([37], but see [38]). Few studies have tested the effect of habitat connectivity on wild bees so far and none of them found an effect of habitat connectivity on wild bees [39], [24]. This might be because these studies did no detailed trait analyses, although e.g. habitat specialists are expected to react stronger to reduced connectivity than habitat generalists [37]. Futheremore, habitat specialists should be influenced more by local habitat area than by landscape composition [40]. Another study also showed that solitary bees show stronger response to habitat area than social bees, but social Halictidae did show stronger response to habitat area than solitary Halictidae [24]. Therefore, traits like trophic rank, habitat specialization, sociality and size should be considered as they have been shown to disentangle factors affecting pollinator diversity [22], [40]–[43].

In this study we aimed to test the relative importance of local factors (habitat area and quality) and landscape factors (habitat connectivity, landscape composition and configuration) of calcareous grasslands for wild bee diversity and different traits of wild bees. We selected 23 calcareous grassland with gradients of local and landscape factors and recorded their bee communities during one season. We expected bee diversity in calcareous grasslands to be influenced by both, local and landscape factors, but traits like habitat specialists or large social species (bumblebees) were expected to differ in their response to local and landscape effects.

## Materials and Methods

### Study region

**Figure 1:**
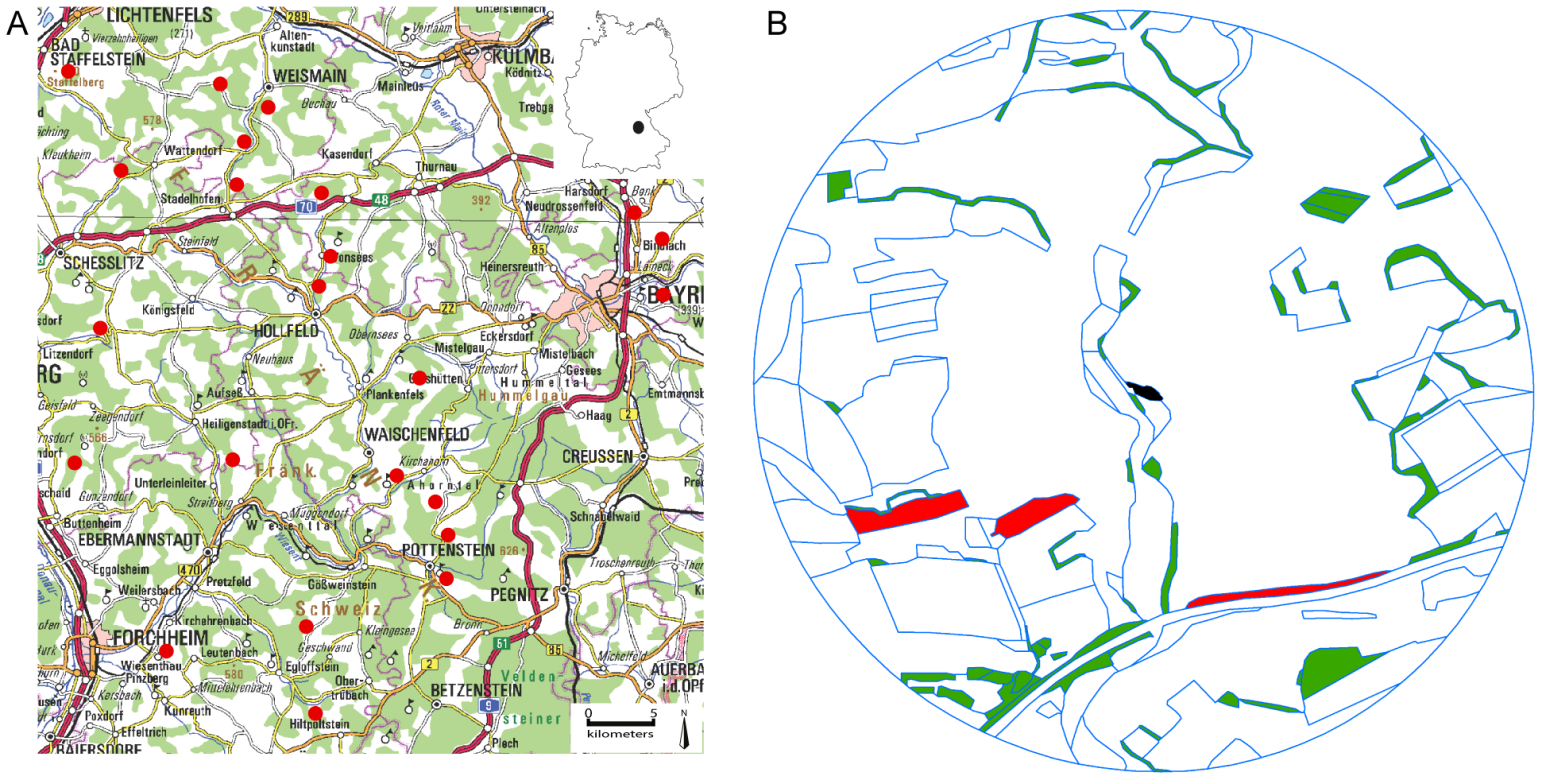
Overview of the study region and example site for illustration of the used landscape metrics. (A) Study region with all sampled sites (red dots). (B) Example site where the black patch in the middle is the sampled calcareous grassland, red patches are calcareous grasslands in the surrounding landscape, green patches are other semi-natural habitats and blue lines are borders between different land-use patches. The Connectivity Index takes area and distance of red patches to the black patch (sampled site) into account. Landscape composition is the percentage of semi-natural habitats (all green and red patches). Landscape configuration is the number of patches (blue lines) in the landscape (patch density).

The study was conducted in Upper Franconia, north-eastern Bavaria, Germany (see Fig. 1). The study region is characterized by its geology, which consists mainly of Jurassic limestone forming a hilly lowland plateau. The total extent of the study region was 45×50 km with altitude varying between 350 and 585 m a.s.l. Mean annual precipitation varies between 650 and 900 mm. The current land use in this area is predominantly characterized by a small-scaled mosaic of arable land, forest, meadows and semi-natural habitats. Important semi-natural habitats are calcareous grasslands that are characteristic for the region and mostly located on hillsides of small valleys.

### Study sites and local habitat parameters

For this study 23 calcareous grasslands were chosen that ranged in size from 0.2 to 11.8 ha. All grasslands were characterized by high flower diversity to reduce for potential effects of flower resources. Flowering plant species excluding graminaceous and tree species were recorded during transect walks (description see section “bees”) and flower cover was estimated. The effect of number of flowering plant species and flower cover (i.e. flower units/ha) on both abundance and species richness of wild bees was tested using simple linear regressions, and did not show significant results (p-values>0.1). Therefore, we focused on habitat quality in terms of nesting resources and choose habitat slope which is a factor that can influence nest density of bees [44], [45] and which is easy to measure. As most bee species in central Europe are soil nesting and direct counting of nesting resources is difficult, we used habitat slope as a simplified factor that influences nesting resources [45]. All grasslands were regularly sheep-grazed (minimum once a year) but two were mown in the end of summer. The effect of historical land-use could not be investigated but might have an influence on current species composition. Habitat area was calculated in ArcGIS 9.3 [46] using orthorecticified digital aerial photos from 2008 with a resolution of 0.2 m (provided by Bayerische Vermessungsverwaltung). As the investigated grasslands were quite homogenous in microstructure, the slope angle did not differ much within the whole grassland. Therefor we used average habitat slope which was calculated in ArcGIS 9.3 [46] using digital contour line maps (provided by Bayerische Vermessungsverwaltung).

### Landscape parameters

Landscape parameters were calculated for landscape sectors with 1 km radius around the patch edges of the grasslands. Based on aerial photos (provided by Bayerische Vermessungsverwaltung), land-use was mapped in the field in August–September 2010. Patches had to be larger than 100 m^2^ to be included in the mapping. Digitizing of field mapping and calculation of landscape parameters were done in ArcGIS 9.3 [46]. We calculated three landscape parameters (see also Fig. 1): (1) the percentage of semi-natural habitats as a measure of landscape composition, where we focused on important bee habitats, i.e. calcareous grasslands, orchards, fallows, ruderal areas, plant species-rich margins and hedgerows. (2) The patch density of the landscape as a measure of landscape configuration, was calculated as the number of all land-use patches in the landscape divided by total landscape area (in km^2^). (3) The habitat connectivity of calcareous grasslands as a measure taking configuration and area of calcareous grasslands in the landscape into account. The habitat connectivity was calculated as Connectivity Index (CI) developed by Hanski [47]. This index has been shown to be a good predictor for species loss of habitat specialists [37], because it takes distance and area of surrounding habitat patches (in this study calcareous grasslands) into account and is described by the following equation:
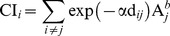
where A*_j_* is the size (in m^2^) of the surrounding habitat patch *j* and d*_ij_* the distance (in km) from the patch to the central focus patch *i*. The parameter α sets the survival rate of migrants as 1/*average migration distance* in km, whereas b scales the size of the habitat patches. According to literature α = 1 was chosen as 1 km appears to be an average dispersal range for wild bees [48]–[50]. For the parameter b a value of 0.5 was chosen as [51] suggested that with increasing patch size, the ratio of patch edge to patch area decreases following A^0.5^. We also calculated the percentage of calcareous grasslands per landscape that was highly correlated with connectivity (r = 0.82), but as it was also correlated with percentage of semi-natural habitats (r = 0.42) we did not use percentage of calcareous grasslands in any analysis.

### Bees

Wild bees (Hymenoptera: Apoidea) were sampled five times from April to August 2010 in “variable transect walks” [52] covering an area of approximately 0.1 ha per study site. Transects had no fixed direction, but were directed to attractive nesting and food resources for bees, whose position could change from month to month. Sampling was conducted from 10.30 to 17.00 h in April and May and from 9.30 to 17.30 in June, July and August. Sampling was only conducted when the temperature was at least 16°C with low wind and sunny weather. Within each transect walk all bees (except honey bees) were caught with a net during a 45 min. period with 9 subunits of 5 min. All individuals that could be identified in the field were recorded and released, otherwise they were stored in ethylacetate and brought to the lab for further identification. Sampling time was stopped during notations or handling of the caught bees. Permissions for sampling of bees and access to protected areas were given by the government of Upper Franconia.

All individuals were identified to species level. Species that were difficult to determine or very rare ones were sent to a specialist for identification. Number of individuals and number of species determined for each study site represented the sum of all five transect walks conducted on that site.

**Figure 2:**
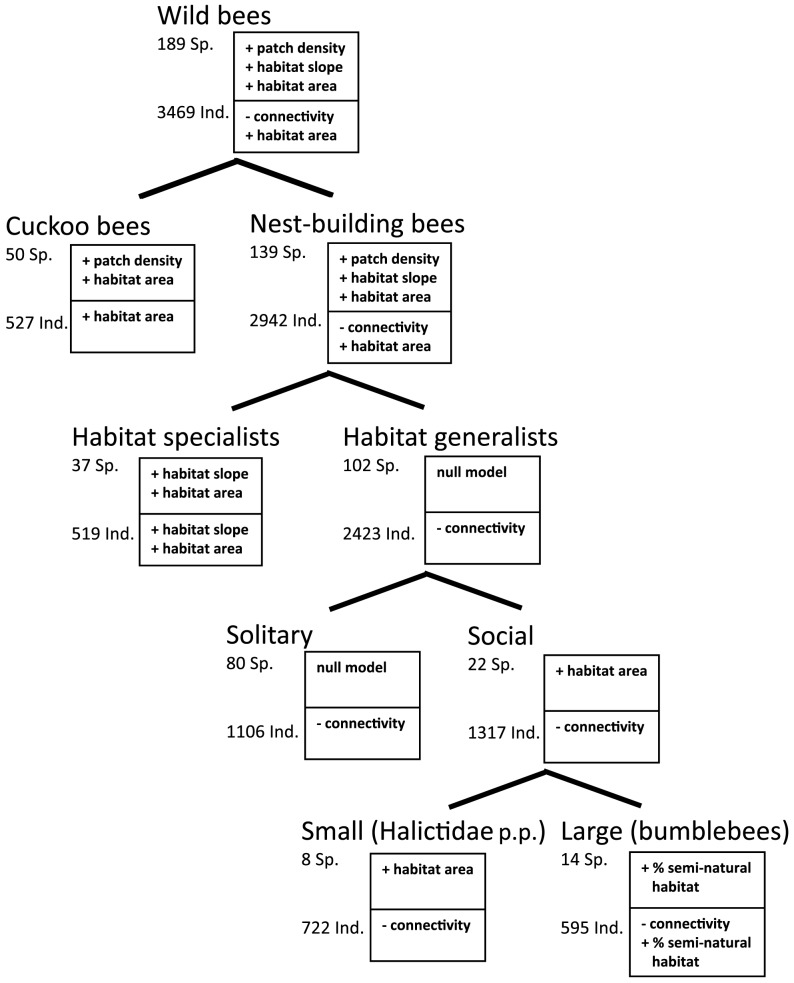
Overview of tested bee-traits. Number of species (Sp.) and of individuals (Ind.) are given for each group and factors that significantly affect them (+ and − indicate the relation of the effects).

As wild bees are expected to show trait specific responses to habitat loss and fragmentation [40], [42], [53], species were grouped according to their life-history traits: we separated cuckoo bees, habitat specialists, solitary bees and small (Halictidae p.p.) and large (bumblebees) social bees according to Westrich [54] (see Fig. 2). Habitat specialization does not represent a real life-history trait [19] but as it is an important characteristic in wild bees we here use the term trait also for habitat specialization. The majority of cuckoo bees were solitary habitat generalists and habitat specialists were almost exclusively solitary species. The complete bee species list with abundance, frequency and trait category can be found in Table S1.

### Statistical analysis

Statistical analyses were performed in R version 2.13.0 [55]. Habitat and landscape effects on bees were tested with general linear models (GLM). The response variables were species richness and abundance and met the assumptions of normality and homoscedasticity [56]. The predictor variables were connectivity (CI), percentage of semi-natural habitats (arcsin√p-transformed to increase linearity in proportion data, see [57]), patch density, habitat slope and habitat area (log_10_-transformed to increase linearity, see [58]). Predictor variables were not inter-correlated in Spearman rank correlation tests (all |R|≤0.35, see [59]). Model fit was checked following Zuur et al. [56]. Full models were manually simplified in backward steps beginning with the highest non-significant p-values from F-tests with type 1 sums of squares. This was done until only significant (p<0.05) variables were left in the model.

To check if two trait categories (e.g. cuckoo bees and nest-building bees) react different to a predictor variable we compared the slopes of regression lines using linear mixed effects models (lme). If the GLMs revealed a significant effect of a predictor variable (e.g. patch area) on both of the dichotomous trait categories, we tested in an lme whether the interaction between predictor and trait category was significant, i.e. whether the slope of regression lines differed for different trait categories. Fixed factors were the predictor of interest (patch area, patch density or connectivity), trait category and the interaction between the predictor of interest and trait category. Response variables were the richness or abundance of the bees within the dichotomous trait categories. Study site was included as random factor.

To check for sufficiency of sampling, first-order jack-knife estimates of species richness were calculated for every grassland patch (with pooled survey rounds) and for the total study region using EstimateS version 9 [60]. Mean observed bee species richness was 67% (range: 62–72%) of estimated species richness of the grassland patches and 83% of the total study region. Estimated sampling sufficiency was not related to habitat area (F = 0.28, p = 0.599) nor did estimated species richness change the results of analyses compared to observed species richness of the grassland patches.

As bees might have large dispersal distances we also checked for spatial autocorrelation.

Therefore we calculated Bray-Curtis similarity of bee communities between all site pairs in EstimateS [60] and spatial distance between all site pairs in ArcGIS 9.3 [46]. We tested the similarity matrix for spatial autocorrelation using a Mantel test (package vegan in R [55]) and found no significant spatial autocorrelation (p = 0.142).

## Results

In total, 3469 wild bee individuals of 189 species belonging to 25 genera were collected on the 23 calcareous grasslands, representing 55% of the wild bee species occurring in Upper Franconia [61]. In Figure 2 the number of individuals and of species are given for all tested groups of wild bees. A total of 35 species were endangered according to the Red List of Bavaria [62] and most of those were habitat specialists.

### Local factors

**Figure 3:**
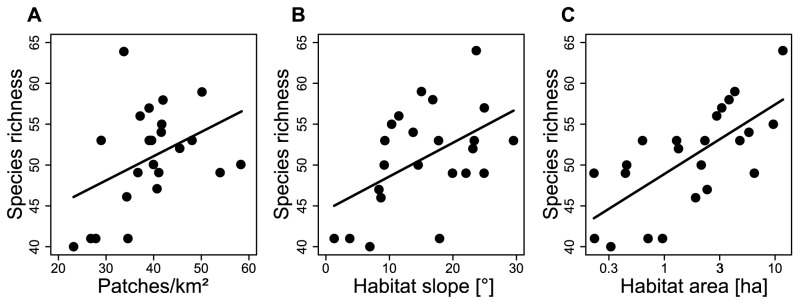
Factors affecting total wild bee richness. Relationship between patch density (A), habitat slope (B) and habitat area (C) and total wild bee richness. Regression lines: (A) y = 0.29x+39.20, (B) y = 0.41x+44.47, (C) y = 3.69x+48.89.

Habitat area of the calcareous grasslands strongly affected wild bees: species richness and abundance of total wild bees, of cuckoo bees and of nest-building bees increased with increasing habitat area (Table 1, Fig. 2 and 3). Within the group of nest-building bees, species richness and abundance of habitat specialists, but not of total habitat generalists, increased with increasing habitat area. Within the group of total habitat generalists, only the abundance of small social generalists (Halictids) increased with increasing habitat area (resulting also in an increase of total social generalists), while large social generalists alone (bumblebees) and solitary generalists were not affected by habitat area. Cuckoo bees did not show a stronger response to habitat area than nest-building bees as the slopes of the regression lines were not significantly different (interaction term in lme with p>0.1).

**Table 1:** Results of general linear models.

Response variables	Predictors	d.f.	F value	P
Total wild bee richness	Patch density (+)	1,19	14.3	0.001
	Habitat slope (+)	1,19	11.9	0.003
	Habitat area (+)	1,19	42.0	<0.001
Total wild bee abundance	Connectivity (−)	1,20	13.0	0.002
	Habitat area (+)	1,20	9.8	0.005
Nest-building bee richness	Patch density (+)	1,19	5.6	0.028
	Habitat slope (+)	1,19	13.2	0.002
	Habitat area (+)	1,19	17.3	<0.001
Nest-building bee abundance	Connectivity (−)	1,20	11.2	0.003
	Habitat area (+)	1,20	7.1	0.015
Cuckoo bee richness	Patch density (+)	1,20	6.5	0.020
	Habitat area (+)	1,20	18.4	<0.001
Cuckoo bee abundance	Habitat area (+)	1,21	5.1	0.034
Habitat generalist richness	Null model	1,22	-	-
Habitat generalist abundance	Connectivity (−)	1,21	16.5	<0.001
Habitat specialist richness	Habitat slope (+)	1,20	16.5	<0.001
	Habitat area (+)	1,20	19.8	<0.001
Habitat specialist abundance	Habitat slope (+)	1,20	6.5	0.020
	Habitat area (+)	1,20	7.3	0.014
Social generalist richness	Habitat area (+)	1,21	6.5	0.019
Social generalist abundance	Connectivity (−)	1,21	13.5	0.001
Solitary generalist richness	Null model	1,22	-	-
Solitary generalist abundance	Connectivity (−)	1,21	7.0	0.016
Small social generalist richness	Habitat area (+)	1,21	8.4	0.009
Small social generalist abundance	Connectivity (−)	1,21	6.5	0.019
Large social generalist richness	% semi-natural habitat (+)	1,21	8.5	0.008
Large social generalist abundance	Connectivity (−)	1,20	7.7	0.014
	% semi-natural habitat (+)	1,20	23.3	<0.001

Effects of connectivity, patch density, percentage semi-natural habitats, patch slope and patch area on abundance and richness of different groups of wild bees. (+) and (−) indicate the relation of the effects.

Steep slopes of the grasslands positively affected species richness and abundance of habitat specialists (resulting also in a positive effect on the species richness of nest-building species and of total wild bees), but did neither effect cuckoo bees nor any of the habitat generalist bee groups (Table 1, Fig. 3).

### Landscape Factors

**Figure 4:**
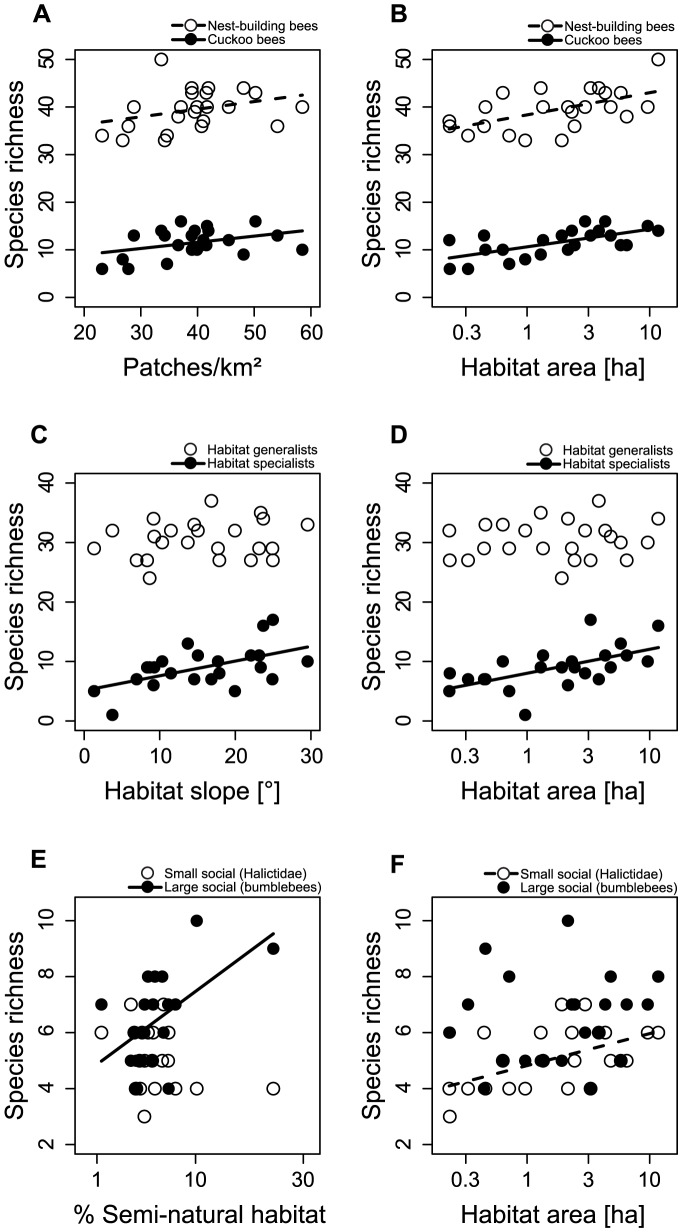
Local and landscape factors affecting different bee traits. Effects of patch density (A) and habitat area (B) on nest-building and cuckoo bees. Effects of habitat slope (C) and habitat area (D) on habitat specialists and generalists. Effects of percentage semi-natural habitat (E) and habitat area (F) on small and large social generalists. Regression lines of significant relationships: (A) Nest-building bees: y = 0.16x+33.15; Cuckoo bees: y = 0.13x+6.37; (B) Nest-building bees: y = 4.66x+38.35; Cuckoo bees: y = 3.70x+10.62; (C) Habitat specialists: y = 0.25x+5.11; (D) Habitat specialists: y = 4.03x+8.02; (E) Large social: y = 11.32x+3.73; (F) Small social: y = 1,14x+4,82.

Landscape configuration affected total wild bees, cuckoo bees and nest-building bees: the richness of these groups was higher in landscapes with higher patch density (number of patches in 1 km radius around the grasslands) (Table 1, Fig. 2–4). Cuckoo bees and nest-building bees did not differ in the strength of their response to landscape configuration (interaction term in lme with p>0.1).

Landscape composition only affected the species richness and abundance of large social habitat generalists (bumblebees), which increased with increasing percentage of semi-natural habitat (Table 1). The gradient in percentage of semi-natural habitats showed one very low and two high values that clearly separate in the graphical plot of bumblebee richness and percentage of semi-natural habitats (Fig. 4e). Therefore we calculated additional models, but removing any of the three data points did not remove the significant effect of percentage of semi-natural habitats (F_1,20_ = 5.1, p = 0.035; F_1,20_ = 6.0, p = 0.024; F_1,20_ = 12.3, p = 0.002) and removing all three data points still resulted in a marginally not significant effect (F_1,18_ = 3.6, p = 0.074).

**Figure 5:**
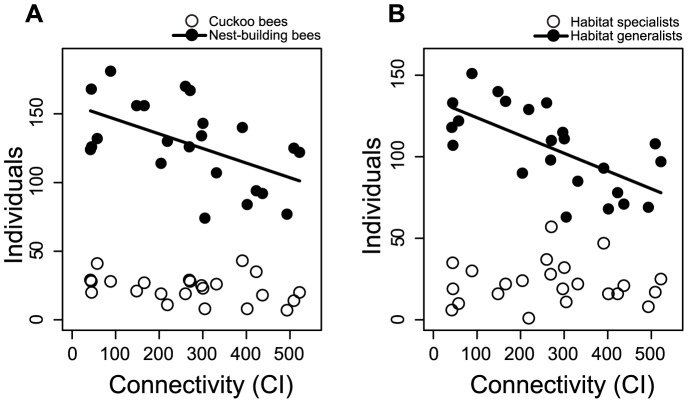
Effects of connectivity on wild bee abundance. Effects of connectivity on nest-building bees and cuckoo bees (A) and on habitat specialists and generalists (B). Regression lines: (A) y = −0,11x+156,67; (B) y = −0,11x+134,84.

High connectivity of the calcareous grasslands did not show the expected positive effect on habitat specialists, but had a negative effect on the abundance of all groups except on cuckoo bees and habitat specialists (Table 1, Fig. 5). Solitary and social as well as small and large social bees did not differ in the strength of their response to connectivity (interaction term in lme with p>0.1).

## Discussion

In our study, we investigated the effects of local and landscape factors on bee communities in calcareous grasslands. We could show that the relative importance of these factors differs among bee groups with different combinations of life-history traits: habitat specialists were affected by local factors only, small social habitat generalists and cuckoo bees were affected by both local and landscape factors, and solitary habitat generalists and large social habitat generalists were affected by landscape factors only.

### Local Factors

Habitat specialists, cuckoo bees and small social generalists showed a positive species-area relationship. The positive relationship between species richness and habitat area is a common and often shown pattern in ecology [26], [63]. However, different species groups may react differentially, for example specialized bees and higher trophic levels are expected to be more sensitive to habitat loss than generalists [41], [42]. The positive species-area relationship for small social generalists but not for solitary and large social generalists is in accordance with results of Öckinger et al. [40]. Social species need large amounts of resources within their foraging range to provide food to a large number of larvae and as all social Halictidae have a quite short foraging distance compared to the large social bumblebees [64] they are expected to be more dependent on local than on landscape factors. Resource concentration as described by Root [65] might also influence species richness and abundance as large calcareous grasslands provide diverse food and more abundant and nesting resources especially for habitat specialists and a variety of host species for cuckoo bees.

Species richness and abundance of habitat specialists increased with increasing habitat slope. This coincides with our expectations that habitat specialists highly depend on the quality of their habitat. Habitat slope influences habitat quality of calcareous grasslands as steep slopes promote erosion and bare soil and increase solar radiation on south exposed slopes compared to flat areas. Potts and Willmer [44] showed that the nest density of the ground-nesting bee *Halictus rubicundus* was positively related with slope angle of the nesting sites. As most of the central European bees are ground nesting [14], bare soil is an essential factor for diverse bee communities [30]. High soil temperature and low soil humidity might be a more important factor for nesting sites of calcareous grassland specialist than for generalists. Nevertheless, there is still a huge lack of knowledge if and how nesting site availability can regulate bee communities [63].

### Landscape Factors

We found landscape configuration i.e. patch density as an important factor for total wild bee richness, and both cuckoo bee and nest-building bee richness. Patch density increases the amount of edges and corridors that can act as food and nesting resource like hedgerows, field margins and ditches [36], [66], [67] and promote dispersal [35]. There is still a lack of knowledge how important landscape composition and configuration are for animal diversity [17]. A recent meta-analysis pointed out the importance of landscape composition but not configuration for wild bees in agroecosystems [12]. This seems contrasting to our findings, but Kennedy et al. [12] analyzed wild bee diversity in crop systems that provide foraging resources but mostly no nesting resources like calcareous grasslands do. This might explain the different results because crop systems need certain amounts of semi-natural habitats in their surroundings to be visited by a variety of pollinators [12], [68] but high quality bee habitats should profit of a highly structured landscape with a variety of other habitats especially linear habitats like ditches and forest edges, which host additional species [54], [66]. We found that cuckoo bees showed a positive response to patch density like nest-building bees but not a stronger one. Higher trophic levels like cuckoo bees are expected to react stronger to habitat fragmentation than their hosts [38] but there are also studies showing the opposite [24], [58]. There is almost no knowledge if nest site fidelity and dispersal distances of cuckoo bees differ from their hosts, but as they have no nesting site they should be more mobile. As most cuckoo bee species have a wide host range [54] they might be not strongly dependent on local habitat quality, but might profit of dispersal corridors provided by structurally rich landscape.

We found no relationship between the percentage of semi-natural habitat in the surrounding landscape and either total species richness or abundance. The large social bumblebees were the only group showing a relationship in which richness as well as abundance increased with increasing percentage of semi-natural habitats. Bumblebees that have a high dispersal capacity [49] seemed to be promoted by large amounts of food resources at a landscape scale [69]. Williams et al. [70] showed that colony growth of bumblebees was driven by flower resources on a landscape scale and thus stable flower resources like semi-natural habitats should enhance abundance of bumblebees. As semi-natural habitats like orchards or calcareous grasslands also provide a variety of nesting cavities [54] the species richness of bumblebees should also be enhanced by the amount of these habitats.

In contrast to our expectations, habitat specialists did not benefit from high habitat connectivity. We found decreasing abundances of all groups except cuckoo bees and habitat specialists, with increasing connectivity. This effect might be the result of a concentration of bees on strongly fragmented grasslands as described in “the landscape-moderated concentration and dilution hypothesis” [71]. Bees that nest in the landscape (like habitat generalists and bumblebees) should also be found on calcareous grasslands as these provide a stable food resource. Our results suggest that in landscapes with high connectivity between the grasslands, foraging bees dilute and disperse on several grasslands whereas in landscapes with low connectivity bees concentrate on a few grassland patches. This implies that bees, especially generalists profit from nearby calcareous grasslands and highly connected landscapes could have positive effects on the fitness of wild bees due to shorter foraging distances. Effects and importance of habitat connectivity differ among recent studies [28], [37], [40], [72] and are therefore controversially discussed [15], [16], [73]. In contrast to butterflies or plants [37], [71] the species richness of bees has not yet been shown to be influenced by habitat connectivity in studies that tested this factor [39], [40], [74]. The Connectivity Index was developed to explain butterfly movement and dispersal in fragmented landscapes [47] and was shown to be good predictor of habitat specialized butterflies [37]. For wild bees connectivity seemed to influence the foraging behavior leading to a dilution in highly connected landscapes.

The portion of local and landscape effects explaining species richness and abundance of the different species groups varied in this study. Habitat specialists were only affected by local factors whereas cuckoo bees and bumble bees were predominantly influenced by landscape configuration and composition, respectively. Structurally rich landscapes with low land-use intensity can accommodate a diverse bee fauna and are therefore important target areas for conservation. But such landscapes are more and more altered to homogeneous landscapes and hence important factors influencing bee communities have to be considered for providing stable ecosystems in the future. According to our results we conclude that large and high-quality habitats are important for diverse bee communities. However, landscape configuration enhanced total wild bee richness and landscape composition at least bumblebee richness and abundance. This implies that structure and quality of agricultural landscapes are also of importance. Decision makers in ecosystem service planning and conservation should therefore strongly promote and restore areas that include both large high-quality habitats and landscapes with high configurational complexity.

## Supporting Information

Table S1 Total species list of wild bees.
(DOC)
