# Supplementary material for: Trait-Specific Responses of Wild Bee Communities to Landscape Composition, Configuration and Local Factors
Source: PLoS One. 2014 Aug 19;9(8):e104439. doi: 10.1371/journal.pone.0104439 (PMC4138035; doi:10.1371/journal.pone.0104439)
Supplement: Table S1 — Total species list of wild bees. (DOC) [file pone.0104439.s001.doc]

**Table S1 Total species list of wild bees.** Abundance, frequency (number of grasslands where a species was found) and trait category for 189 bee species found on 23 grasslands.

| **Family** | **Species** | **Abundance** | **Frequency** | **Trait category** |
| --- | --- | --- | --- | --- |
| Andrenidae | *Andrena barbilabris* | 1 | 1 | Solitary habitat generalists |
| Andrenidae | *Andrena bicolor* | 3 | 3 | Solitary habitat generalists |
| Andrenidae | *Andrena chrysosceles* | 11 | 6 | Solitary habitat generalists |
| Andrenidae | *Andrena cineraria* | 47 | 15 | Solitary habitat generalists |
| Andrenidae | *Andrena combinata* | 6 | 4 | Habitat specialist |
| Andrenidae | *Andrena curvungula* | 1 | 1 | Habitat specialist |
| Andrenidae | *Andrena falsifica* | 57 | 15 | Solitary habitat generalists |
| Andrenidae | *Andrena flavipes* | 157 | 22 | Solitary habitat generalists |
| Andrenidae | *Andrena fucata* | 1 | 1 | Solitary habitat generalists |
| Andrenidae | *Andrena fulva* | 1 | 1 | Solitary habitat generalists |
| Andrenidae | *Andrena fulvago* | 2 | 2 | Solitary habitat generalists |
| Andrenidae | *Andrena fulvata* | 4 | 3 | Solitary habitat generalists |
| Andrenidae | *Andrena gravida* | 9 | 7 | Solitary habitat generalists |
| Andrenidae | *Andrena haemorrhoa* | 25 | 13 | Solitary habitat generalists |
| Andrenidae | *Andrena hattorfiana* | 3 | 2 | Solitary habitat generalists |
| Andrenidae | *Andrena helvola* | 5 | 4 | Solitary habitat generalists |
| Andrenidae | *Andrena humilis* | 1 | 1 | Solitary habitat generalists |
| Andrenidae | *Andrena intermedia* | 1 | 1 | Habitat specialist |
| Andrenidae | *Andrena jacobi* | 6 | 4 | Solitary habitat generalists |
| Andrenidae | *Andrena labialis* | 4 | 2 | Solitary habitat generalists |
| Andrenidae | *Andrena labiata* | 9 | 6 | Solitary habitat generalists |
| Andrenidae | *Andrena minutula* | 12 | 6 | Solitary habitat generalists |
| Andrenidae | *Andrena minutuloides* | 19 | 10 | Solitary habitat generalists |
| Andrenidae | *Andrena nigroaenea* | 20 | 12 | Solitary habitat generalists |
| Andrenidae | *Andrena nitida* | 15 | 10 | Solitary habitat generalists |
| Andrenidae | *Andrena ovatula* | 51 | 19 | Habitat specialist |
| Andrenidae | *Andrena pandellei* | 2 | 2 | Solitary habitat generalists |
| Andrenidae | *Andrena proxima* | 12 | 9 | Solitary habitat generalists |
| Andrenidae | *Andrena strohmella* | 9 | 5 | Solitary habitat generalists |
| Andrenidae | *Andrena subopaca* | 8 | 7 | Habitat specialist |
| Andrenidae | *Andrena tibialis* | 1 | 1 | Solitary habitat generalists |
| Andrenidae | *Andrena vaga* | 3 | 3 | Solitary habitat generalists |
| Apidae | *Anthophora aestivalis* | 13 | 7 | Habitat specialist |
| Apidae | *Anthophora furcata* | 1 | 1 | Solitary habitat generalists |
| Apidae | *Anthophora plumipes* | 3 | 3 | Solitary habitat generalists |
| Apidae | *Anthophora quadrimaculata* | 3 | 3 | Solitary habitat generalists |
| Apidae | *Bombus hortorum* | 17 | 10 | Large social habitat generalist |
| Apidae | *Bombus humilis* | 39 | 16 | Large social habitat generalist |
| Apidae | *Bombus hypnorum* | 1 | 1 | Large social habitat generalist |
| Apidae | *Bombus jonellus* | 1 | 1 | Large social habitat generalist |
| Apidae | *Bombus lapidarius* | 172 | 23 | Large social habitat generalist |
| Apidae | *Bombus lucorum* | 6 | 2 | Large social habitat generalist |
| Apidae | *Bombus pascuorum* | 137 | 23 | Large social habitat generalist |
| Apidae | *Bombus pratorum* | 33 | 17 | Large social habitat generalist |
| Apidae | *Bombus ruderarius* | 1 | 1 | Large social habitat generalist |
| Apidae | *Bombus soroeensis* | 29 | 11 | Large social habitat generalist |
| Apidae | *Bombus subterraneus* | 1 | 1 | Large social habitat generalist |
| Apidae | *Bombus sylvarum* | 47 | 11 | Large social habitat generalist |
| Apidae | *Bombus terrestris agg* | 98 | 21 | Large social habitat generalist |
| Apidae | *Bombus wurflenii* | 13 | 9 | Large social habitat generalist |
| Apidae | *Ceratina cyanea* | 22 | 11 | Habitat specialist |
| Apidae | *Epeolus variegatus* | 8 | 6 | Cuckoo bee |
| Apidae | *Melecta albifrons* | 1 | 1 | Cuckoo bee |
| Apidae | *Melecta luctuosa* | 3 | 2 | Cuckoo bee |
| Apidae | *Nomada alboguttata* | 1 | 1 | Cuckoo bee |
| Apidae | *Nomada bifasciata* | 20 | 13 | Cuckoo bee |
| Apidae | *Nomada conjungens* | 4 | 4 | Cuckoo bee |
| Apidae | *Nomada distinguenda* | 1 | 1 | Cuckoo bee |
| Apidae | *Nomada emerginata* | 1 | 1 | Cuckoo bee |
| Apidae | *Nomada fabriciana* | 5 | 4 | Cuckoo bee |
| Apidae | *Nomada ferruginata* | 1 | 1 | Cuckoo bee |
| Apidae | *Nomada flava* | 9 | 7 | Cuckoo bee |
| Apidae | *Nomada flavoguttata* | 27 | 12 | Cuckoo bee |
| Apidae | *Nomada flavopicta* | 1 | 1 | Cuckoo bee |
| Apidae | *Nomada fucata* | 23 | 10 | Cuckoo bee |
| Apidae | *Nomada goodeniana* | 25 | 11 | Cuckoo bee |
| Apidae | *Nomada integra* | 1 | 1 | Cuckoo bee |
| Apidae | *Nomada lathburiana* | 19 | 12 | Cuckoo bee |
| Apidae | *Nomada marshamella* | 18 | 13 | Cuckoo bee |
| Apidae | *Nomada piccioliana* | 1 | 1 | Cuckoo bee |
| Apidae | *Nomada ruficornis* | 6 | 4 | Cuckoo bee |
| Apidae | *Nomada sexfasciata* | 4 | 2 | Cuckoo bee |
| Apidae | *Nomada sheppardana* | 3 | 3 | Cuckoo bee |
| Apidae | *Nomada signata* | 1 | 1 | Cuckoo bee |
| Apidae | *Nomada succincta* | 15 | 7 | Cuckoo bee |
| Apidae | *Psithyrus barbutellus* | 4 | 3 | Cuckoo bee |
| Apidae | *Psithyrus bohemicus* | 37 | 11 | Cuckoo bee |
| Apidae | *Psithyrus campestris* | 5 | 5 | Cuckoo bee |
| Apidae | *Psithyrus norvegicus* | 5 | 3 | Cuckoo bee |
| Apidae | *Psithyrus rupestris* | 10 | 5 | Cuckoo bee |
| Apidae | *Psithyrus sylvestris* | 5 | 5 | Cuckoo bee |
| Apidae | *Thyreus orbatus* | 1 | 1 | Cuckoo bee |
| Colletidae | *Colletes cunicularius* | 4 | 4 | Solitary habitat generalists |
| Colletidae | *Colletes daviesanus* | 7 | 2 | Solitary habitat generalists |
| Colletidae | *Colletes similis* | 2 | 2 | Habitat specialist |
| Colletidae | *Hylaeus annularis* | 6 | 6 | Habitat specialist |
| Colletidae | *Hylaeus brevicornis* | 5 | 3 | Solitary habitat generalists |
| Colletidae | *Hylaeus communis* | 4 | 2 | Solitary habitat generalists |
| Colletidae | *Hylaeus confusus* | 6 | 5 | Solitary habitat generalists |
| Colletidae | *Hylaeus difformis* | 2 | 2 | Solitary habitat generalists |
| Colletidae | *Hylaeus gredleri* | 3 | 2 | Solitary habitat generalists |
| Colletidae | *Hylaeus hyalinatus* | 22 | 10 | Solitary habitat generalists |
| Colletidae | *Hylaeus kahri* | 1 | 1 | Solitary habitat generalists |
| Colletidae | *Hylaeus nigritus* | 5 | 3 | Habitat specialist |
| Colletidae | *Hylaeus paulus* | 2 | 2 | Solitary habitat generalists |
| Colletidae | *Hylaeus sinuatus* | 6 | 4 | Solitary habitat generalists |
| Colletidae | *Hylaeus styriacus* | 3 | 3 | Solitary habitat generalists |
| Colletidae | *Hylaeus variegatus* | 1 | 1 | Habitat specialist |
| Halictidae | *Dufourea dentiventris* | 3 | 2 | Solitary habitat generalists |
| Halictidae | *Dufourea inermis* | 2 | 2 | Solitary habitat generalists |
| Halictidae | *Halictus confusus* | 2 | 1 | Habitat specialist |
| Halictidae | *Halictus eurygnathus* | 1 | 1 | Habitat specialist |
| Halictidae | *Halictus maculatus* | 32 | 6 | Small social habitat generalist |
| Halictidae | *Halictus rubicundus* | 34 | 9 | Small social habitat generalist |
| Halictidae | *Halictus scabiosae* | 18 | 7 | Habitat specialist |
| Halictidae | *Halictus sexcinctus* | 2 | 2 | Solitary habitat generalists |
| Halictidae | *Halictus simplex agg* | 58 | 11 | Habitat specialist |
| Halictidae | *Halictus tumulorum* | 185 | 22 | Small social habitat generalist |
| Halictidae | *Lasioglossum aeratum* | 1 | 1 | Solitary habitat generalists |
| Halictidae | *Lasioglossum albipes* | 27 | 12 | Solitary habitat generalists |
| Halictidae | *Lasioglossum calceatum* | 196 | 23 | Small social habitat generalist |
| Halictidae | *Lasioglossum costulatum* | 15 | 7 | Solitary habitat generalists |
| Halictidae | *Lasioglossum fratellum* | 11 | 9 | Solitary habitat generalists |
| Halictidae | *Lasioglossum fulvicorne* | 152 | 21 | Solitary habitat generalists |
| Halictidae | *Lasioglossum laevigatum* | 3 | 3 | Solitary habitat generalists |
| Halictidae | *Lasioglossum laticeps* | 31 | 14 | Small social habitat generalist |
| Halictidae | *Lasioglossum lativentre* | 14 | 11 | Solitary habitat generalists |
| Halictidae | *Lasioglossum leucopus* | 6 | 5 | Habitat specialist |
| Halictidae | *Lasioglossum leucozonium* | 31 | 16 | Solitary habitat generalists |
| Halictidae | *Lasioglossum lineare* | 2 | 2 | Habitat specialist |
| Halictidae | *Lasioglossum lissonotum* | 1 | 1 | Habitat specialist |
| Halictidae | *Lasioglossum malachurum* | 3 | 1 | Habitat specialist |
| Halictidae | *Lasioglossum minutulum* | 6 | 6 | Habitat specialist |
| Halictidae | *Lasioglossum morio* | 134 | 23 | Small social habitat generalist |
| Halictidae | *Lasioglossum nitidulum* | 29 | 12 | Solitary habitat generalists |
| Halictidae | *Lasioglossum parvulum* | 6 | 5 | Habitat specialist |
| Halictidae | *Lasioglossum pauxillum* | 108 | 18 | Small social habitat generalist |
| Halictidae | *Lasioglossum punctatissimum* | 3 | 2 | Solitary habitat generalists |
| Halictidae | *Lasioglossum puncticolle* | 2 | 2 | Small social habitat generalist |
| Halictidae | *Lasioglossum pygmaeum* | 1 | 1 | Habitat specialist |
| Halictidae | *Lasioglossum quadrinotatum* | 1 | 1 | Solitary habitat generalists |
| Halictidae | *Lasioglossum villosulum* | 48 | 15 | Solitary habitat generalists |
| Halictidae | *Lasioglossum xanthopus* | 2 | 1 | Solitary habitat generalists |
| Halictidae | *Lasioglossum zonulum* | 3 | 2 | Solitary habitat generalists |
| Halictidae | *Rophites algirus* | 8 | 1 | Habitat specialist |
| Halictidae | *Sphecodes albilabris* | 3 | 3 | Cuckoo bee |
| Halictidae | *Sphecodes crassus* | 10 | 6 | Cuckoo bee |
| Halictidae | *Sphecodes ephippius* | 86 | 22 | Cuckoo bee |
| Halictidae | *Sphecodes ferruginatus* | 29 | 12 | Cuckoo bee |
| Halictidae | *Sphecodes geofrellus* | 1 | 1 | Cuckoo bee |
| Halictidae | *Sphecodes gibbus* | 15 | 9 | Cuckoo bee |
| Halictidae | *Sphecodes hyalinatus* | 20 | 10 | Cuckoo bee |
| Halictidae | *Sphecodes monilicornis* | 56 | 18 | Cuckoo bee |
| Halictidae | *Sphecodes pellucidus* | 1 | 1 | Cuckoo bee |
| Halictidae | *Sphecodes puncticeps* | 10 | 8 | Cuckoo bee |
| Halictidae | *Sphecodes reticulatus* | 2 | 2 | Cuckoo bee |
| Halictidae | *Sphecodes rufiventris* | 6 | 1 | Cuckoo bee |
| Megachilidae | *Anthidium manicatum* | 6 | 5 | Solitary habitat generalists |
| Megachilidae | *Anthidium oblongatum* | 3 | 2 | Habitat specialist |
| Megachilidae | *Anthidium punctatum* | 25 | 11 | Habitat specialist |
| Megachilidae | *Anthidium strigatum* | 6 | 5 | Habitat specialist |
| Megachilidae | *Chelostoma campanularum* | 17 | 9 | Solitary habitat generalists |
| Megachilidae | *Chelostoma distinctum* | 1 | 1 | Solitary habitat generalists |
| Megachilidae | *Chelostoma florisomne* | 1 | 1 | Solitary habitat generalists |
| Megachilidae | *Chelostoma rapunculi* | 7 | 5 | Solitary habitat generalists |
| Megachilidae | *Coelioxys afra* | 7 | 4 | Cuckoo bee |
| Megachilidae | *Coelioxys conica* | 3 | 3 | Cuckoo bee |
| Megachilidae | *Coelioxys elongata* | 2 | 2 | Cuckoo bee |
| Megachilidae | *Coelioxys mandibularis* | 4 | 4 | Cuckoo bee |
| Megachilidae | *Heriades truncorum* | 15 | 8 | Solitary habitat generalists |
| Megachilidae | *Megachile alpicola* | 2 | 2 | Solitary habitat generalists |
| Megachilidae | *Megachile centuncularis* | 3 | 3 | Solitary habitat generalists |
| Megachilidae | *Megachile circumcincta* | 4 | 2 | Habitat specialist |
| Megachilidae | *Megachile ericetorum* | 1 | 1 | Habitat specialist |
| Megachilidae | *Megachile nigriventris* | 6 | 5 | Solitary habitat generalists |
| Megachilidae | *Megachile pilidens* | 15 | 8 | Habitat specialist |
| Megachilidae | *Megachile versicolor* | 5 | 5 | Solitary habitat generalists |
| Megachilidae | *Megachile willughbiella* | 1 | 1 | Solitary habitat generalists |
| Megachilidae | *Osmia adunca* | 22 | 6 | Habitat specialist |
| Megachilidae | *Osmia andrenoides* | 5 | 1 | Habitat specialist |
| Megachilidae | *Osmia aurulenta* | 60 | 19 | Habitat specialist |
| Megachilidae | *Osmia bicolor* | 125 | 21 | Solitary habitat generalists |
| Megachilidae | *Osmia brevicornis* | 6 | 4 | Solitary habitat generalists |
| Megachilidae | *Osmia caerulescens* | 1 | 1 | Solitary habitat generalists |
| Megachilidae | *Osmia claviventris* | 5 | 4 | Solitary habitat generalists |
| Megachilidae | *Osmia leaiana* | 1 | 1 | Solitary habitat generalists |
| Megachilidae | *Osmia leucomelana* | 9 | 8 | Solitary habitat generalists |
| Megachilidae | *Osmia mitis* | 7 | 4 | Habitat specialist |
| Megachilidae | *Osmia rufa* | 23 | 10 | Solitary habitat generalists |
| Megachilidae | *Osmia rufohirta* | 43 | 11 | Habitat specialist |
| Megachilidae | *Osmia spinulosa* | 60 | 13 | Habitat specialist |
| Megachilidae | *Osmia uncinata* | 3 | 2 | Solitary habitat generalists |
| Megachilidae | *Osmia xanthomelana* | 9 | 6 | Habitat specialist |
| Megachilidae | *Stelis ornatula* | 1 | 1 | Cuckoo bee |
| Megachilidae | *Stelis punctulatissima* | 5 | 4 | Cuckoo bee |
| Megachilidae | *Stelis signata* | 1 | 1 | Cuckoo bee |
| Megachilidae | *Trachusa byssina* | 31 | 13 | Habitat specialist |
| Melittidae | *Melitta haemorrhoidalis* | 10 | 7 | Solitary habitat generalists |
| Melittidae | *Melitta leporina* | 1 | 1 | Solitary habitat generalists |
